# Manual Wheelchair Use: Bouts of Mobility in Everyday Life

**DOI:** 10.1155/2012/753165

**Published:** 2012-07-15

**Authors:** Sharon Eve Sonenblum, Stephen Sprigle, Ricardo A. Lopez

**Affiliations:** Rehabilitation Engineering and Applied Research Laboratory, Georgia Institute of Technology, 490 10th Street NW, Atlanta, GA 30318, USA

## Abstract

*Background*. This study aimed to describe how people move about in manual wheelchairs (MWCs) during everyday life by evaluating bouts of mobility or continuous periods of movement. *Methods*. A convenience sample of 28 MWC users was recruited. Participants' everyday mobility was measured using a wheel-mounted accelerometer and seat occupancy switch for 1-2 weeks. Bouts of mobility were recorded and characterized. *Results*. Across 29,200 bouts, the median bout lasted 21 seconds and traveled 8.6 m at 0.43 m/s. 85% of recorded bouts lasted less than 1 minute and traveled less than 30 meters. Participants' daily wheelchair activity included 90 bouts and 1.6 km over 54 minutes. Average daily occupancy time was 11 hours during which participants wheeled 10 bouts/hour and spent 10% of their time wheeling. Spearman-Brown Prophecy analysis suggested that 7 days were sufficient to achieve a reliability of 0.8 for all bout variables. *Conclusions*. Short, slow bouts dominate wheelchair usage in a natural environment. Therefore, clinical evaluations and biomechanical research should reflect this by concentrating on initiating movement, maneuvering wheelchairs, and stopping. Bouts of mobility provide greater depth to our understanding of wheelchair use and are a more stable metric (day-to-day) than distance or time wheeled.

## 1. Introduction

The study of activity has been of interest for many years as a means to relate activity and health outcomes. The study of activity specifically among persons with disabilities has garnered recent interest with respect to health and community participation [1, 2]. Decreased mobility can impact health status and has been associated with issues such as diabetes and obesity [3–6].

As a means to characterize activity, research has documented how far people walk daily [7–9], and guidelines have been developed to establish goals or metrics for walking activity [10, 11]. Bohannon synthesized published data and documented similar walking activity across gender, and differences across certain geographic regions [12]. Furthermore, he found that most studies reported that adults, especially older adults, in the United States walked fewer than the 10,000-step criterion.

Analogous data has also been collected on manual wheelchair mobility with authors reporting the distance traveled over a day and sometimes reporting the amounts of time spent moving and average speed [13–17].  Table 1 lists the results of five such studies. Despite diverse subject groupings, the daily distance results are fairly similar with the exception of a study using competing athletes.

Other research into mobility considered *how people move* as opposed to *how far people move*. Bouts of mobility, or continuous segments of movement, have been reported as a means to describe ambulation and wheelchair movement [6–8, 18, 19]. In ambulation studies, steps taken over short epochs of time are reported as a means to describe walking patterns [6, 7]. Results indicated that people overwhelmingly walk in short bursts. Levine reported that 97% of ambulation bouts lasted less than 200 seconds, and Orendurff et al. reported 90% lasted less than 100 steps.

Bouts of wheelchair mobility have been measured and reported for power wheelchair users [19]. When applying this construct to wheeled mobility, bouts of mobility reflect volitional transitions between functional activities and are defined by a combination of distance traveled and minimum velocity. Bouts of powered wheelchair movement mimic the reported ambulation data in that most bouts were short in distance and duration. 69% of bouts were shorter than 30 seconds and traversed less than 7.6 m.

A fuller understanding of how people use manual wheelchairs can benefit a variety of stakeholders. Wheelchair users and clinicians are obvious stakeholders that would benefit from relating wheelchair use to wheelchair selection. Manufacturers can use information about how their products are used to impact the design process and to tailor different designs to different patterns of use. Finally, entities that pay for wheelchairs would benefit from a better understanding of how wheelchair equipment is used across demographic and environmental factors.

This study's objective was to describe how people move about in manual wheelchairs during everyday life by evaluating bouts of mobility. Information about bouts of movement will be presented alongside traditional measures of use to illustrate its ability to enhance the understanding of manual wheelchair usage.

## 2. Methods

### 2.1. Participants

A convenience sample of 28 adults who used manual wheelchairs as their primary mobility devices were recruited for this study with IRB approval. Because this was a descriptive study, the sample size was not based upon power to discern differences, but reflected practical concerns (i.e., available time and resources). Data collection continued until additional subject data no longer changed our outcome metrics of typical wheelchair use. Participants were primarily recruited through a rehabilitation hospital. Reflective of the hospital's patient mix, the majority of participants had a SCI diagnosis, but an effort was made to recruit participants with different diagnoses as well. Subjects signed informed consent forms prior to beginning their participation in the study.

### 2.2. Instrumentation & Protocol

**Figure 1:**
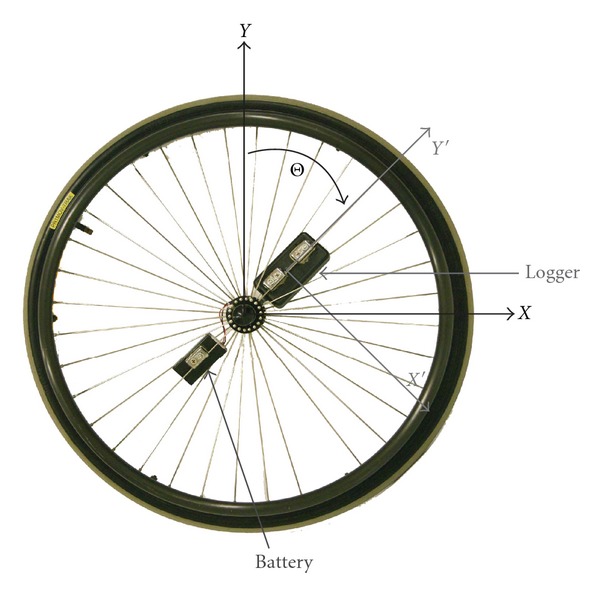
Spoked MWC wheel instrumented with the data logger and its corresponding battery pack. The data logger axes (X′ and Y′), oriented radially and tangentially, respectively, are parallel to the wheel plane (X and Y). Only this plane was analyzed. Acceleration along the third axis, perpendicular to the wheel plane, was not used.

Each participant's wheelchair was instrumented with a data logging system. The data logging system features a solid-state, triaxial, micro-electro-mechanics system (MEMS) accelerometer with a ±2 g range at its core (Freescale MMA7260Q) connected to a data logging system built around Microchip's PIC18LF2331 microcontroller. The logging system is comprised of the logger box (10 cm × 5 cm × 3 cm, 120 grams) and the battery pack (6.8 cm by 3.5 cm by 1.8 cm, 100 grams), which holds 2 standard alkaline AA cells. Battery life allowed for up to 2 weeks of continuous logging. For a complete description of the electronics, including instructions for constructing the system, please see http://rearlab.gatech.edu/wheelchair_data_logger.php. Acceleration was sampled at a rate of 10 Hz, based on earlier validation work [20]. The logging system was mounted on the right wheel, as seen in Figure 1, for periods between 1 and 2 weeks (depending on subject availability).

Wheelchair occupancy was measured using a mechanical switch (AliMed Chair Sensor Pad) placed under the wheelchair cushion and logged through the external analog input of a second data logger (MSR145). Occupancy was sampled every 5 seconds. Finally, subjects were asked to complete a survey containing standard demographic questions and information about wheelchair use.

### 2.3. Data Analysis

Data processing was performed using Matlab R2010B, and statistical analyses were done using Minitab 15. Detailed methods for using acceleration to determine if the wheelchair is moving, the distance wheeled, and the wheeling velocity have been presented elsewhere [20], but a brief description of the processing of the accelerations is described below.

The two orthogonal axes of acceleration coplanar with the wheel (X′ and Y′ in Figure 1) were filtered through a 2nd order low-pass Butterworth filter with a cutoff frequency of 3.1 Hz (this includes wheeling speeds of up to 3 m/s in the filtered data). Next the inverse tangent of the ratio of the two filtered accelerations (unwrapped) determined the wheel's rotation angle. The derivative of rotation angle multiplied by the wheel circumference provided the wheelchair velocity. The velocity was filtered using a 2nd order low-pass Butterworth with a 0.5 Hz cutoff and compared with a threshold velocity. Velocities in either direction greater than 0.12 m/s were considered to be moving. This method of measuring manual wheelchair movement offers a rate of accuracy greater than 90% across manual wheelchair models, wheel type (mag and spoke), speeds, and indoor and outdoor surfaces [20].

The velocity, distance, and time data were used to calculate bouts of mobility. A bout of mobility is defined as a volitional transition between activities. To be consistent with earlier work [19], a bout was defined as any wheelchair movement that (i) lasted at least 5 seconds, (ii) had a speed greater than or equal to 0.12 m/s, and (iii) ended when less than 0.76 m were wheeled within 15 seconds.

The third bout criterion implies that very brief stops or periods of exceptionally slow movement may be included within a larger bout.

Once bouts of mobility were identified, statistics about their duration, distance, and speed were gathered. Daily bout statistics were also computed, including only full days of data collection. The days on which equipment installation and removal occurred were excluded. Occupancy data was summarized across subject days, and the average time spent in the wheelchair (occupancy time) was reported. Additionally, the percentage of time in the wheelchair spent wheeling (% mobile) and the number of mobility bouts wheeled per hour spent in the wheelchair (bout frequency) were calculated. Day-to-day variation is presented in terms of the coefficient of variation (CV). The CV was computed within each subject for each variable and averaged across all subjects. To better understand how the metrics of wheelchair usage relate, correlations were calculated between the metrics and Pearson correlation coefficients are reported. Finally, to support future studies on wheelchair usage, we analyzed the minimum number of days needed to provide a good estimate of daily wheelchair usage. The Spearman-Brown Prophecy Formula [21] was used to predict the number of days required to achieve acceptable reliability (alpha = 0.80 and 0.90). Multiple days of data for 17 subjects (mobility metrics) and 14 subjects (occupancy metrics) were used to calculate the estimated reliability used in the Spearman-Brown Prophecy Formula. One-way ANOVAs were also run to identify any differences across days of the week or between weekdays and weekends.

## 3. Results

### 3.1. Subjects

28 adults, ages from 22 to 67 years old (median 34.5) participated in this study. All used manual wheelchairs as their primary means of mobility and propelled with their upper extremities. Participants included 21 men and 7 women who had been using a wheelchair for an average of 9 years (range 1.5–36 years). 27 participants used an ultralightweight wheelchair, while only 1 used a standard manual wheelchair. Additional descriptions are presented in Table 2.

### 3.2. Bouts of Mobility

**Figure 2:**
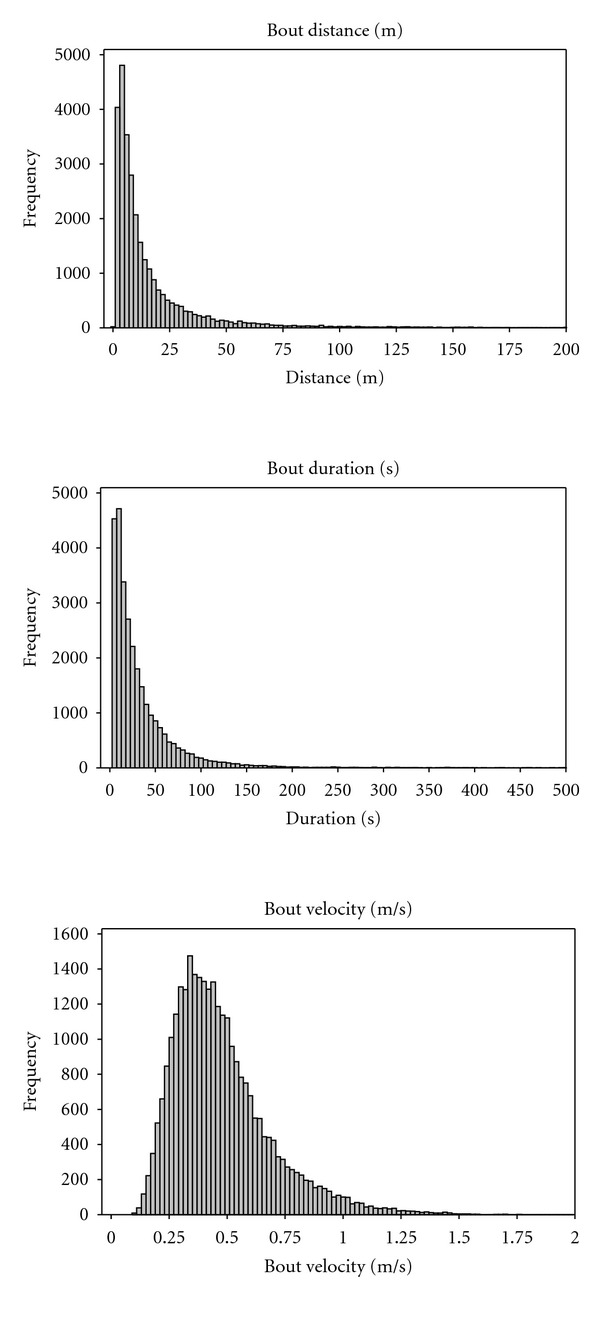
Histograms of typical bout parameters: distance, duration, and velocity.

A total of 29,255 bouts were identified from 370 subject days of data, which included 296 hours and 595 km of total aggregated wheeling. The median bout lasted 21 seconds and traveled 8.6 m at 0.43 m/s (Table 3). The relatively large differences between the mean and median values are indicative of a skewed distribution (Figure 2). Bout velocity was more normally distributed. The median time between bouts was 95 seconds.

Given the skewed distribution of distance and duration, percentiles can be more useful in depicting the data compared to the use of parametric descriptors. For example, 63% of bouts were shorter than or equal to 30 seconds, while 85% of bouts lasted 60 seconds or less in duration. With respect to distance and velocity, 63% of bouts were less than or equal to 12.5 m and occurred at less than 0.5 m/s, and 85% were less than or equal to 30 m and occurred at less than 0.68 m/s. Finally, nearly half of bouts were followed by less than 90 seconds of rest.

### 3.3. Daily Wheelchair Use

The analysis of daily wheelchair use incorporated 278 full subject days. Occupancy measurements were successfully collected on 21 participants and included 201 days of data. Hardware failures excluded the use of the remaining days. The median daily occupancy was 11.2 hours, ranging from 11 minutes to 24 hours. The median daily wheelchair user traversed 1.6 km over 54 minutes, broken up into 90 bouts of mobility (Table 4). On a typical day, participants were wheeling for approximately 10% of the time they were seated in their wheelchairs and completed approximately 10 bouts per hour they were seated in their wheelchairs. Finally, day-to-day variation was considerable for all variables, with occupancy time exhibiting the least day-to-day variation. 

Pearson correlation coefficients indicated significant relationships between most metrics of daily mobility (Table 5). The daily distance was very highly correlated with the time spent wheeling on the same day. Bouts per day showed a slightly stronger relationship with time moving compared to distance. Occupancy time had weaker relationships with distance, time moving, and bouts of mobility. The percent of time spent moving also had weaker relationships with distance and time moving and no relationship with the number of bouts. Occupancy time and percent of time moving was negatively correlated, as was occupancy time and bout frequency. 

Results of the Spearman-Brown Prophecy analysis suggested that 4 days were sufficient to achieve a reliability of 0.8 for all variables except the percent mobile, which required 7 days (Table 6). An increased reliability of 0.9 required up to 9 days for all variables except percent mobile which required 15 days. Additionally, ANOVA tests revealed no differences between days of the week or between weekdays and weekends.

## 4. Discussion

This study reports how people move about in manual wheelchairs using bouts of mobility. Short, slow bouts dominate wheelchair usage, with approximately 63% of bouts being shorter than 30 seconds and 13 m, and slower than 0.5 m/s. These results highlight the importance of inertia-changing activities such as starting, stopping, and maneuverability, as opposed to long continuous bouts of movement. Because changes in inertia require more force than maintaining velocity, wheelchair propulsion research should include inertial changes to gain a more complete picture of propulsion efficiency. Moreover, stresses on the upper extremity will be greater during acceleration compared to constant velocity movement which indicates a need to study wheelchair mechanics during starting, stopping and turning [22]. The predominance of short bouts of movement also informs clinical prescription of wheelchairs. Concentration on initiating movement, maneuvering wheelchairs, and stopping are key operational parameters that should be emphasized during wheelchair evaluation.

The dominance of short bouts in manual wheelchair use is consistent with walking patterns in able-bodied adults [7]. Orendurff et al. found a very similar distribution of bouts in a population of employed adults (Table 7). This suggests that manual wheelchair users move like typical Americans. On the other hand, when you consider the total amount of movement, able-bodied adults in the United States were found to take approximately 7,000 steps per day [12] which corresponds to more than 5 km per day—considerably more than wheelchair users in the current study.

The median and mean bout speed was 0.43 m/s and 0.48 m/s, respectively. These velocities are comparable to the values reported in the literature, which range from 0.48 to 0.80 m/s [13–17]. These values are less than both self-selected gait and wheelchair propulsion speeds [23, 24]. These slower speeds are consistent with the short bouts of mobility, and by extension, the conclusion that slow bouts are endowed with maneuvering the wheelchair. The normal distribution of bout velocity (Table 3) suggests that users appear to have a preferred speed during everyday mobility, and parametric analysis can be used to discern differences across groups or interventions.

We also found that bouts appear to be clustered together, with approximately half of the bouts separated by less than 90 seconds from the next bout. This has implications for activity levels and the required endurance for wheelchair use. The data suggest that long, steady-state wheeling is uncommon, so sufficient endurance necessary to be a successful wheelchair user may be embodied by the endurance required for repeatedly initiating and completing short bouts of movement without a sufficient recovery period.

Wheelchair use varied widely across and within subjects (Table 4), consistent with previous work on power wheelchairs [19]. Interestingly, occupancy and the daily number of bouts show less within subject day-to-day variability than distance and duration. Because the number of bouts reflects transitions between activities, consistency in daily bouts of movement may be reflective of a relatively consistent number of activities performed, such as activities of daily living and daily routines, compared to distance and time that have greater volatility. Additionally, fewer days of measurement are required to describe an individual's typical wheelchair use in terms of number of bouts and occupancy time, as compared with bout distance and duration. The great variability across subjects indicates that the amount of propulsion forces exerted by wheelchair users also varies widely, and that the duration of wheelchair use does not accurately reflect the magnitude of cumulative stressors on the body.

Moreover, the smaller correlations between some of the metrics of wheelchair use mean that those metrics are measuring different constructs. This finding was consistent with the relationships between metrics used to describe powered mobility usage [19]. In that study, we illustrated how two people can travel the same distance with different numbers of bouts and durations of travel. The same result was found with MWC users. In summary, using different metrics to characterize wheelchair use can paint a fuller picture of wheeled mobility.

### 4.1. Study Limitations

One limitation of this study was that, despite efforts to recruit a broad population, 71% of enrolled participants had a diagnosis of SCI, and only two participants had diagnoses not associated with spinal cord dysfunction. Insufficient data was collected to determine if individuals with other diagnoses would have different behavior, but it is likely that factors such as age and comorbidities would influence wheelchair use and behaviors. Another limiting factor in our dataset is environment. All data was collected in one US city which impacts the means by which people travel in the community and the size, design, and layout of individuals' homes. Other studies have found age, race, and employment status [16, 17] to influence overall wheelchair use although they did not consider the influence on bouts of mobility. The data set should be expanded to include a more diverse population and different geographical regions to determine which factors have the greatest influence on bouts of mobility.

## 5. Conclusions

Daily MWC usage varies widely across and within people. This variability is not adequately represented by reporting global metrics such as total distance and time wheeled. In particular, the bout of mobility serves to improve the description of mobility used during everyday activities. More research is needed to combine the mostly quantitative approach described in this work with demographics and environmental factors. This will be useful to identify relevant factors influencing wheelchair use that can help in the prescription, funding, and design of wheelchairs.

## Figures and Tables

**Table 1:** Five studies measuring manual wheelchair use in everyday life.

Study	Population	Daily distance	Daily time moving	Daily average speed
Karmarkar et al. [14]	VA nursing homes	1.5 km	n/a	0.48 m/s
Levy et al. [15]	Adults	1.45 km	n/a	n/a
Tolerico et al. [17]	Athletes	2.5 km	48 min	0.8 m/s
Cooper et al. [13]	Children	1.6 km	n/a	0.67 m/s
Oyster et al. [16]	SCI	1.9 km	47 min	0.63 m/s

Ranges	—	1.5–2.5 km	47.5 min	0.5–0.8 m/s

**Table 2:** Subject characteristics.

	Number (%)
Diagnosis	
SCI	20 (71)
Transverse myelitis	4 (14)
Other (CP, spina bifida, spinal cord infarction, ataxia)	4 (14)
Ambulation ability	
Nonambulatory	18 (64)
Able to stand	3 (11)
Able to ambulate at least 2 steps	5 (18)
Do not know	2 (7)
Race	
African-American	6 (21)
Caucasian	18 (64)
Hispanic/Latino	2 (7)
Other	2 (7)
Employment	
Employed	11 (39)
Unemployed	10 (36)
Student	3 (11)
Other or missing	4 (14)
Education	
High school diploma or GED	9 (32)
Associates degree	4 (14)
Bachelors degree	8 (29)
Graduate degree	5 (18)
Other	2 (7)

**Table 3:** Typical bout parameters. The minimum bout duration is 5 seconds by definition.

Bout metric	Mean (SD)	Median (min–max)
Distance (m)	20 (58)	8.6 (0.8–3829.5)
Duration (sec)	36 (61)	21 (5–2419)
Velocity (m/s)	0.48 (0.21)	0.43 (0.09–1.98)

**Table 4:** Daily wheelchair use.

Daily metric	Mean (SD)	Median (min–max)	Day-to-day CV (%)
Daily distance (km)	1.953 (1.525)	1.617 (0.007–10.472)	50
Daily time moving (min)	58.1 (37.6)	54.3 (0.5–208.1)	43
Bouts per day	96 (50)	90 (3–235)	33
Occupancy time (hours)	10.5 (5.2)	11.2 (0.2–24.0)	28
% Mobile (%)	11.2 (8.8)	9.3 (0.4–56.0)	44
Bouts per occupancy hour	11 (6)	10 (1–31)	32

**Table 5:** Correlations were high between daily distance and time spent wheeling, but lower when comparing metrics with number of bouts or occupancy time. Pearson correlation coefficient (*P*-value).

	Daily time moving	Bouts per day	Occupancy time	% Mobile	Bout frequency
Daily distance	0.932 (0.000)	0.665 (0.000)	0.488 (0.000)	0.227 (0.001)	0.058 (0.416)
Daily time moving	1	0.819 (0.000)	0.476 (0.000)	0.292 (0.000)	0.174 (0.014)
Bouts per day		1	0.558 (0.000)	0.035 (0.626)	0.195 (0.006)
Occupancy time			1	−0.489 (0.000)	−0.530 (0.000)
% Mobile				1	0.787 (0.000)

**Table 6:** Number of days needed for desired levels of reliability (*α*) differed across variables.

Variable	*α* = 0.8	*α* = 0.9
Bout distance	3	7
Bout duration	2	5
Number of bouts	1	3
Occupancy time	1	3
Percent mobile	7	15
Bout frequency	4	9

**Table 7:** Comparison of typical bouts in walking (Orendurff et al. [7]) compared with manual wheelchair use.

Walking	Manual WC
60% ≤ 30 s	63% ≤ 30 s
81% ≤ 70 s	85% ≤ 60 s
